# Food Choice, Nutrition, and Public Health

**DOI:** 10.3390/foods14071243

**Published:** 2025-04-02

**Authors:** Mariana Santos, Ricardo Assunção

**Affiliations:** 1 Food and Nutrition Department, National Institute of Health Doutor Ricardo Jorge, 1649-016 Lisbon, Portugal; rassuncao@egasmoniz.edu.pt; 2 Comprehensive Health Research Center (CHRC), Universidade NOVA de Lisboa, 1600-560 Lisbon, Portugal; 3 Egas Moniz Center for Interdisciplinary Research (CiiEM), Egas Moniz School of Health & Science, Caparica, 2829-511 Almada, Portugal

Maintaining a healthy diet throughout life helps prevent all forms of malnutrition, thereby reducing the risk of non-communicable diseases (NCDs) and related conditions [1].

Dietary habits, lifestyle choices, and social factors significantly influence our health. Recently, we have seen shifts in food consumption patterns driven by various factors. These include the increased availability of processed foods, rapid urbanization, and changing lifestyles [2].

The types, amounts, and frequency of consumed foods and beverages define dietary patterns, which have been evolving in recent decades due to the emergence of new or adapted eating habits. Common examples of dietary patterns include the ‘Western dietary pattern’ and the ‘Mediterranean dietary pattern’. Other significant patterns are the ‘prudent dietary pattern’, which emphasizes a high intake of vegetables, fruits, legumes, whole grains, fish, and seafood, and the ‘vegetarian/plant-based dietary pattern’, which entirely omits meat and animal products [3].

Understanding the individual motives that drive certain food choices is crucial for changing consumption habits, promoting healthier behaviors, and fostering sustainability [4].

This Editorial introduces the Special Issue “Food Choice, Nutrition, and Public Health” and highlights key topics on this subject.

***Dietary Behavior, Public Health, and Policy***

Effective government policies and actions are key to creating healthy food environments that enable healthy diets and reduce the prevalence of obesity, diet-related noncommunicable diseases (NCDs), and related inequalities. Food environments are recognized as having a major influence on people’s diets. Public policies have the significant potential to help create a healthy food environment that encourages people to eat well [5].

Food choices and dietary behavior are complex and highly variable, influenced by various psychological, social, cultural, biological, and economic factors. The growing quantity and variety of available food products have resulted in consumer choices becoming increasingly complex, and this has also affected individuals’ environments [6].

Food choice contributes to the health status of consumers, with particular impacts for certain food components, for instance, salt intake, as is discussed in Contribution 1. In this context, and regarding the knowledge, attitudes, and behaviors (KABs) related to dietary salt, Turkish authors recently assessed KABs in Turkey, according to gender and blood pressure determinations, in terms of predictors of salt-related dietary behaviors. Canyolu and Sadıç [7] concluded that the majority of participants (*n* = 415) were aware of the health risks associated with high salt intake. These authors also concluded that higher overall attitude scores increased the odds of performing behaviors with the aim of reducing salt, except for checking food labels, using spices instead of salt, and purchasing foods labeled as low sodium. Despite this, salt-related KABs were not found to be aligned with positive attitudes toward salt consumption, thus highlighting the need for regulations that operate independently of public perception, such as policies for reducing salt in the food industry and making reduced-salt products more affordable to encourage healthier food choices.

Contribution 2 evaluated the eating habits of a sample population of 995 adults from Southern Italy (601 participants) and from the Dominican Republic (394 participants), focusing on fruit and vegetable (FV) consumption and its association with skin carotenoid levels, as measured with the Veggie Meter^®^. Authors found that the Italian sample had a higher frequency of consumption of fruits and vegetables in their main meals (lunch and dinner) and had higher carotenoid scores than the Dominican population. Applying multiple linear regression analysis, Augimeri et al. [8] found that the carotenoid score was positively associated with sex, age, vegetable consumption, and the perception of a healthy diet in the Dominican sample and was directly associated with sex and both vegetable and fruit consumption in the Italian sample.

Contribution 3 investigated the pattern of beverage consumption in different groups of Spanish individuals (aged between 18 and 45 years) and its relationship with other nutritional variables and habits through an observational, descriptive, and cross-sectional study. The main results showed significant differences in beverage consumption patterns between the socio-demographic variables of sex, age, and educational level, as well as between different regions of Spain. Principal component analysis (PCA) showed a relationship between the population’s sugar-sweetened beverage consumption and the Healthy Diet Index and physical activity. Sandri et al. [9] concluded that the population’s beverage consumption pattern was influenced by socio-demographic variables and that healthier drinking habits affect the nutrition and health of the population.

Contribution 4 evaluated the impact of household income level on dietary imbalance among older rural-dwelling individuals, aged 65 and above (*n* = 3614), and also explored the heterogeneity of household income structure and its role in the relationship between the two. Scientific evidence regarding the relationship between income and food prices with intakes was described previously [10]. Focusing the elderly people, Wang et al. [11] investigated the impact of household income level on dietary imbalances among the elderly rural-dwelling individuals. The authors verified that an increase in total household income significantly improves the dietary quality of elderly rural-dwelling individuals and the income structure variable enhances its negative effect on dietary imbalance. The authors concluded that it is necessary to increase the non-working income of the elderly, strengthen social responsibility for their care, and alleviate the problem of dietary imbalance among older rural-dwelling individuals.

***Nutrition’s Role in Disease Prevention and Health Outcomes***

The role of healthy nutrition and diet in the prevention of NCDs, namely a high intake of fruit and vegetables (FVs), has consistently been associated with a reduced risk of several chronic diseases [12]. Furthermore, the abundance of unprocessed plant foods typical of all well-planned diets, including vegetarian diets, can provide the body with numerous protective factors [13,14].

Contribution 5 conducted a narrative review to provide an up-to-date analysis of systematic reviews and meta-analyses published in the past five years (2018–2023), dealing with the effects of FV consumption on human health, focusing on specific pathologies, such as total mortality, cancer, cardiovascular diseases (CVDs), type 2 diabetes, intestinal inflammation, and bone and respiratory illnesses. Devirgiliis et al. [15] assessed a total of 28 publications. The results confirmed the protective role of FV in preventing NCDs, particularly CVDs. However, the study emphasized the need for further evidence and clarifications of the impact of confounding factors by conducting more randomized control trials and using more standardized approaches and designs. These findings could help establish a relationship between FV consumption and human health.

Contribution 6 aimed to highlight the main benefits of vegetarian diets on the risk and course of the most common NCDs. Baroni et al. [16] investigated the health benefits of vegetarian diets through a meta-analysis of observational and intervention studies. The results found that an abundance of non-processed plant foods, including vegetarian ones, can provide the body with numerous protective factors (fiber, phytocompounds), while limiting the intake of harmful nutrients like saturated fats, heme-iron, and cholesterol. The beneficial effects of this balance on health have been reported for many chronic diseases, in both observational and intervention studies.

In Contribution 7, a meta-epidemiological assessment based on systematic reviews was carried out in order to examine existing clinical trials that investigated the effects of the ketogenic diet on patients (aged > 18 years) with obesity and diabetes between 1946 and 2024. A total of seven studies encompassing 161 participants were considered. While the macronutrient content of a ketogenic diet explicitly utilized for childhood epilepsy is clearly defined in the literature, variations in other ketogenic diets exhibit substantial heterogeneity. Trials using ketogenic diets include several confounding factors with significant effects on outcomes, making both their results and those of the meta-analyses less reliable. The results of the meta-epidemiological assessment highlight the significant confounding factors in randomized control trials (RCTs) that affect the reliability of current meta-analyses. These are mainly due to inconsistent adherence to defined macronutrient ratios, the inadequate monitoring of nutritional ketosis, and the influence of starvation conditions in very low-calorie ketogenic diets (VLCKDs). Hunter et al. [17] concluded that conducting a meta-analysis of these trials is not advisable at this time, as it could lead to misleading conclusions. They recommended that (i) future studies on the effects of ketogenic diets adopt a standardized definition, and (ii) to accurately assess the true effects of a ketogenic diet, it is essential to measure actual macronutrients and calorie intake while regularly monitoring nutritional ketosis.

***Dietary Intake, Body Composition, and Athletic Performance***

Numerous studies have demonstrated that human body composition is influenced by several mechanisms, including diet, physical activity/exercise, and genetic and behavioral factors. Maintaining a healthy, balanced diet in conjunction with regular exercise represents a modifiable strategy for ensuring optimal health throughout the life cycle [18,19].

Contribution 8 aimed to elucidate whether the intake of macronutrients and B vitamins could be associated with the variation in body fat percentage in a cohort of elite Lithuanian female athletes (*n* = 89). The study results revealed that despite a slightly positive energy balance (D 95 kcal/day), the carbohydrate-deficient diet, along with higher intakes of vitamin B1, vitamin B2, and vitamin B3 from food, was associated with a lower percentage of body fat in female athletes. Therefore, Baranauskas et al. [20] concluded that when female athletes with a higher body fat percentage attempt to achieve optimal body composition by limiting carbohydrates, they should be cognizant of potentially lowering their protein and vitamin B intake.

In Contribution 9, Lombardo et al. [21] investigated the effects of the nutritional patterns and physical activity on the body composition of 1342 participants aged 18–65 years. Differences in dietary habits, taste preferences, and the variety of protein sources were also considered. Gender- and age-related differences in weekly food consumption and protein source variety were identified. In general, men exhibited a higher consumption of meat, processed meat and fish than women, especially in younger age groups. Differences between age groups in the consumption of dairy and soy food products were also identified. Physical activity also affects body composition, namely fat mass. Among non-sporting individuals, vegetarians exhibited a lower fat mass, while among athletes, vegetarians who took part in the endurance sports and pescatarians who participated in strength sports exhibited a lower fat mass. The obtained results reinforced the complex interaction between diet, body composition, and lifestyle choices.

***Ethical Considerations and Consumer Behavior***

Eating often involves ethical choices that are rooted in the context of cultures, traditions, and social structures that influence human nutrition and health outcomes in a globalized way. This topic examines how ethics and perceptions influence food choices, especially around meat and plant-based options [22].

Regarding the consumption of red meat, Hou et al. [23], in Contribution 10, explored the use of virtual reality (VR) using an experimental design with 142 participants to understand how participants develop an understanding of the life and death of farm animals and examine the relationship between presence and empathy and to experimentally investigate whether differences in individual empathy levels alter the sense of presence experienced within VR and, in turn, affect attitudes toward meat consumption. The authors reported that in the VR context, individuals with higher empathy scores experienced a stronger sense of presence, significantly influencing their attitudes toward beef consumption, mediated by a change in anti-beef-eating attitude. Thus, VR can serve as a medium to reduce individuals’ willingness to consume beef, despite the study only focusing on the short-term changes in attitudes following the VR intervention. These results reinforce the importance of preventing health risks and consequently impacting public health.

Also related to the consumption of meat products, in Contribution 11, Ciobotaru et al. [24] assessed the nutritional quality of meat products and compared them to their protein-based (PB) alternatives in the United Kingdom, applying different tools as back-of-pack (BoP) and front-of-pack (FoP) nutritional information, the healthiness of the products using nutrient profiling, and reviewing the nutrition and health claims declared on the packaging. Results showed that meat products had higher protein, fat, and saturated fat content, whilst PB alternatives were higher in dietary fiber and carbohydrates; red color coding for fat and saturated fat (‘high’) was more prominent in meat products, and the red meat category had the most products with high fat and saturated fat content; and most red meat PB alternatives made a nutrition claim, all related to the protein content. The authors concluded that PB alternatives can be considered healthier substitutes for meat products, basing their conclusions in nutrition information displayed on food labels that are traditionally used for assessing the healthiness of food products, supporting consumers’ decisions [25].

***Sustainable Nutrition and Food Sources***

Promoting healthy, sustainable diets is important for sustainable food systems. Country-specific nutrition policies should adopt more holistic strategies, encompassing broader issues within nutrition guidelines and policies. This integration is particularly pertinent for national food-based dietary guidelines. Health- and climate-centered dietary guidelines highlight the crucial role of promoting sustainable food consumption, underscoring the interconnection between human and planetary health [26,27]

Knowledge of the amino acid composition of food products could be used by clinicians when specific amounts are required, as suggested by Contribution 12, by Das et al. [28], who assessed the information on the amino acid composition and protein content of commercially important food fish, namely small indigenous fish. These authors concluded that among the fish studied, *Eleotris fusca*, *Macrobrachium malcomsonii*, and *Mystus cavasius* were rich in most of the amino acids important for human nutrition, such as glycine, glutamic acid, cysteine, threonine, phenylalanine, methionine, lysine, leucine, isoleucine, histidine, and valine. The authors also concluded that including nutrient-dense indigenous fish in a regular diet is a good strategy for obtaining animal protein and nutrients in the cheapest way possible, thus contributing to public health.

Another perspective on the importance of minerals food contents was presented by Nascimento et al. [29], in Contribution 13. The authors measured the mineral contents (Cu, Mn, Fe, Zn, Mg, P, Ca, K, and Na) in different pseudocereals, namely quinoa (*Chenopodium quinoa*), amaranth (*Amaranthus caudatus*), and buckwheat (*Fagopyrum esculentum*) and concluded that pseudocereals align with current guidelines for sustainable and plant-based diets, minimizing the intake of processed foods and animal products while promoting environmentally friendly practices like reducing food waste and opting for locally sourced and seasonal ingredients.

In Contribution 14, Fras et al. [30] looked at the procedure for developing new Slovenian Food-Based Dietary Guidelines (SLO FBDG). In February 2023, the Slovenian Strategic Council for Nutrition proposed new Food-Based Dietary Guidelines (FBDGs) integrating health and environmental considerations. The SLO FBDG recommends a predominantly plant-based diet (high intake of vegetables, fruits, whole grains, pulses, potatoes, nuts, and seeds) with moderate intakes of dairy products, eggs, and fish; a limited intake of meat; and minimal (ideally no) intakes of processed meat, alcohol, and processed foods high in saturated fats, salt, refined starch/grains, and added/free sugar. Drinking plain water, mineral water, or non-sweetened tea is recommended as a primary beverage. The FBDGs, centered on health and climate, emphasize the importance of promoting sustainable food consumption, linking the importance of food to human and planetary health.

In Contribution 15, Czarniecka-Skubina et al. [31] explored the role of school meals in ensuring nutrition and food security for children during their growing years. The study was conducted in 24 Polish primary schools participating in the “Junior-Edu-Żywienie (JEŻ) Project”. The authors highlighted that both the school and home environments shape students’ eating behavior, making them essential components from a public health perspective. Schools, in particular, provide an excellent setting for practical nutrition education. However, beyond imparting knowledge, it is vital to ensure a consistent message about nutrition, reinforced by parents, teachers, and, indirectly, the food and meals available at school. The study emphasized that increasing the availability and attractiveness of nutritious food choices enhances students’ opportunities to consume foods and meals aligned with dietary guidelines, ultimately supporting healthier eating habits.

Fifteen papers were accepted for publication and are included in this Special Issue “Food Choice, Nutrition, and Public Health” (eleven research articles, three reviews, and one article classified as other). The contributions are listed below.

## Data Availability

Not applicable.
